# Exploration of Binding Affinities of a 3β,6β-Diacetoxy-5α-cholestan-5-ol with Human Serum Albumin: Insights from Synthesis, Characterization, Crystal Structure, Antioxidant and Molecular Docking

**DOI:** 10.3390/molecules28165942

**Published:** 2023-08-08

**Authors:** Mahboob Alam

**Affiliations:** Department of Safety Engineering, Dongguk University Wise, 123 Dongdae-ro, Gyeongju-si 780714, Gyeongbuk, Republic of Korea; mahboobchem@gmail.com

**Keywords:** diacetoxy-5α-cholestane, crystal structure HSA binding, multi-spectroscopy, DFT

## Abstract

The present study describes the synthesis, characterization, and in vitro molecular interactions of a steroid 3β,6β-diacetoxy-5α-cholestan-5-ol. Through conventional and solid-state methods, a cholestane derivative was successfully synthesized, and a variety of analytical techniques were employed to confirm its identity, including high-resolution mass spectrometry (HRMS), Fourier transforms infrared (FT-IR), nuclear magnetic resonance (NMR), elemental analysis, and X-ray single-crystal diffraction. Optimizing the geometry of the steroid was undertaken using density functional theory (DFT), and the results showed great concordance with the data from the experiments. Fluorescence spectral methods and ultraviolet–vis absorption titration were employed to study the in vitro molecular interaction of the steroid regarding human serum albumin (HSA). The Stern-Volmer, modified Stern-Volmer, and thermodynamic parameters’ findings showed that steroids had a significant binding affinity to HSA and were further investigated by molecular docking studies to understand the participation of active amino acids in forming non-bonding interactions with steroids. Fluorescence studies have shown that compound **3** interacts with human serum albumin (HSA) through a static quenching mechanism. The binding affinity of compound **3** for HSA was found to be 3.18 × 10^4^ mol^−1^, and the Gibbs free energy change (ΔG) for the binding reaction was −9.86 kcal mol^−1^ at 298 K. This indicates that the binding of compound **3** to HSA is thermodynamically favorable. The thermodynamic parameters as well as the binding score obtained from molecular docking at various Sudlow’s sites was −8.2, −8.5, and −8.6 kcal/mol for Sites I, II, and III, respectively, supporting the system’s spontaneity. Aside from its structural properties, the steroid demonstrated noteworthy antioxidant activity, as evidenced by its IC_50_ value of 58.5 μM, which is comparable to that of ascorbic acid. The findings presented here contribute to a better understanding of the pharmacodynamics of steroids.

## 1. Introduction

Cholestane derivatives are important steroid molecules that have several biological functions in the human body. Cholesterol, the primary precursor for cholestane derivatives, is an essential component of cell membranes and is required for the synthesis of hormones and bile acids [1]. Some cholestane derivatives have demonstrated positive effects in the treatment of different diseases [1,2,3,4,5]. However, it is worth noting that elevated blood cholesterol levels could potentially heighten the risk of heart-related illnesses [6,7,8]. Statins stand out among the extensively researched cholesterol derivatives [3,9], given their remarkable efficacy in lowering cholesterol levels in individuals with hypercholesterolemia [10,11] as well as their application in various other treatments [12]. The mechanism of action of statins involves the inhibition of HMG-CoA reductase [10], an enzyme responsible for cholesterol synthesis in the liver. By reducing cholesterol in the blood, statins can reduce the risk of heart disease and stroke [13]. The cholestane skeleton is a rigid, four-ring structure found in cholesterol and other steroid molecules. Introducing heteroatoms, such as nitrogen, oxygen, and sulfur, into the cholestane skeleton can alter the biological activity of these molecules. Estradiol [14,15] is one example of a steroid hormone that is synthesized from cholesterol and contains three hydroxyl groups. Estradiol is crucial for reproductive health and has other effects such as anti-inflammatory and neuroprotective properties. As a prominent protein in blood plasma, human serum albumin (HSA) is essential for the transport of numerous molecules throughout the body [16,17,18]. HSA has different binding sites that interact with various compounds, including fatty acids, hormones, and drugs [19,20]. Its interaction with drugs is crucial, as it affects the distribution, metabolism, and bioavailability. HSA interacts with small-molecule drugs, peptides, and proteins, and its binding affects drug pharmacokinetics and pharmacodynamics [21,22]. It can also alter drugs’ activity and potency by causing conformational changes in the protein that affect its stability [23]. Furthermore, HSA plays a vital role influencing their pharmacokinetics and pharmacodynamics [24]. The investigation of human serum albumin (HSA) and drug interactions has been greatly aided by molecular docking [19,25]. The technique of molecular docking enables the prediction of binding modes and affinity between drugs and HAS [22]. Its related tools facilitate the exploration of stability and conformational alterations within the protein–drug complex. These tools are essential in drug discovery, as they help researchers understand the mechanisms of drug action, improve drug efficacy, and reduce the risk of adverse effects. Researchers have extensively studied the binding affinities of steroidal and nonsteroidal compounds with human serum albumin (HSA) using various techniques such as synthesis, characterization, crystal structure analysis, and molecular docking [26,27,28,29]. These studies have provided valuable insights into the interactions and structural features of these compounds with HSA and have opened up possibilities for therapeutic applications. A novel preclinical approach to drug optimization has been developed using HSA binding affinity as an indicator of changes in the serum half-life (T1/2). In this strategy, scaffold sites of drug candidates not involved in target interactions are altered to proactively identify longer or shorter dosing regimens for humans to complement existing medicinal chemistry efforts. HSA, a major plasma protein, has a significant impact on drug binding, which in turn affects drug delivery, efficacy, and pharmacokinetics. It is also used in clinical settings as a drug delivery system. In this review [21], the properties of drug binding sites within the HSA structure are described, and an overview of drug–HSA interactions is provided. Cholestane derivatives are being investigated by researchers for their ability to bind to HSA in the body, which may provide insight into molecular mechanisms governing drug metabolism and activity [23,30]. The results of these studies contribute to the development of novel drugs with targeted biological activities, potentially leading to therapeutic advances. Microwave irradiation (MWI) was investigated as a means of introducing acetyl functionality to the OH group [31,32,33]. Acetic acid and acetyl chloride were used as a cheaper acetylating reagent in place of acetic anhydride, which has been banned in some countries due to safety concerns. In conventional processes using acetic anhydride, the reaction takes about 1–4 h at high temperatures and requires a distillation step to separate acetic acid formed during the reaction. The MWI method eliminates excess solvent usage during the reaction, leading to safer and more energy-efficient operation. 3β,6β-diacetoxy-5α-cholestan-5-ol was synthesized using both conventional and MWI methods as part of a green synthesis strategy [34]. Both techniques are equally useful; the former is used for macro-level synthesis, and the latter is used for micro-level synthesis. As part of this study, a comprehensive analysis was conducted to reveal the mechanism of binding between synthesized 3β,6β-diacetoxy-5α-cholestan-5-ol (3) and human serum albumin (HSA). To gain a deeper understanding of the interaction between the compound and the protein, spectroscopic methods and molecular docking were used in this study. The synthesized compound **3** obtained by both conventional and green methods was identified by applying physicochemical techniques, Single-X-ray diffraction, and mixed melting points. This study provided new insights into the binding mechanism of 3β,6β-diacetoxy-5α-cholestan-5-ol and HSA. The investigation involved both in-silico and experimental analysis, which provided comprehensive and supportive information regarding the steroid binding process with HSA. Additionally, the DPPH radical scavenging potential of the compound was determined using the 1,1-diphenyl-2-picrylhydrazine (DPPH) assay and compared to standard ascorbic acid.

## 2. Results and Discussion

### 2.1. Chemistry

The synthesis of 3β,6β-diacetoxy-5α-cholestan-5-ol was achieved through two protocols: conventional and nonconventional. The conventional method is more suitable for macro-level synthesis, likely for larger-scale production, while the nonconventional protocol is better suited for micro-level synthesis in the laboratory setting. The novelty of the nonconventional protocol synthesis lies in utilizing a microwave to speed up the reaction, resulting in a shorter reaction time and higher yield. The solid-state procedure streamlines the reaction workup and improves its environmental friendliness. Notably, the melting points of the products obtained through conventional and solid-state methods are comparable, indicating no product degradation with the solid-state approach. Overall, the solid-state synthesis of 3β,6β-diacetoxy-5α-cholestan-5-ol is a novel and efficient method, made valuable by the combination of microwave acceleration and a simplified workup.

An analysis of the synthesized steroidal compounds obtained by solid-state synthesis as well as conventional methods with a melting point of 167–168 °C revealed that it has the molecular composition C_31_H_52_O_5_. A sharp peak with a medium band at 3480 cm^−1^ corresponds to a tertiary OH group undergoing asymmetric stretching vibration and two sets of strong bands in the ranges of 1238–1266 cm^−1^ and 1736–1741 cm^−1^, indicating the presence of two acetyloxy groups in symmetric and asymmetric stretching vibrations, respectively. A band at 1031 cm^−1^, which indicates the presence of a C-O-C bond with asymmetric stretching vibration, was among the distinctive features that could be seen in the infrared (IR) spectrum of acetoxy-steroids. Different signals could be seen in the nuclear magnetic resonance (NMR) spectrum, including a broad multiplet centered at δ 5.37 (1H, dd, C6-αH, equatorial, J = 9.0, 6.0 Hz Hz, 4.9 (1H, m, W1/2 = 18 Hz) for C3-H, axial, A/B ring junction trans), and a sharp singlet at δ 2.24 for the OH group, which vanished when D_2_O was added. Additionally, two distinct singlets were seen at δ 2.07 and δ 2.0, which stood for two acetoxy groups (CH_3_-CO-O-). The C10-CH_3_ and C13-CH_3_ protons were responsible for the signals at δ 1.03 and δ 0.73, respectively, while other methyl protons showed up at δ 0.97 and δ 0.87. ^13^C NMR spectroscopy established the existence of two acetoxy groups in steroid 3. The signals at δ 171.0 and δ 171.3 correspond to the two acetoxy groups, which could be located at the 3-position and 6-position of the steroidal skeleton. Based on the elemental and physical data provided, which correspond to a melting point of 168°, it is possible to positively identify the substance mentioned in the document, which is known as 3β,6β-diacetoxy-5-hydroxy-5α-cholestane and has a reported melting point of 167°. Finally, the single-crystal X-ray crystallography technique was used for confirming the synthesized compound **3** geometry.

### 2.2. X-ray Crystal Structure and Molecular Geometry of the Synthesized 3β,6β-Diacetoxy-5-hydroxy-5α-cholestane (3)

**Figure 1:**
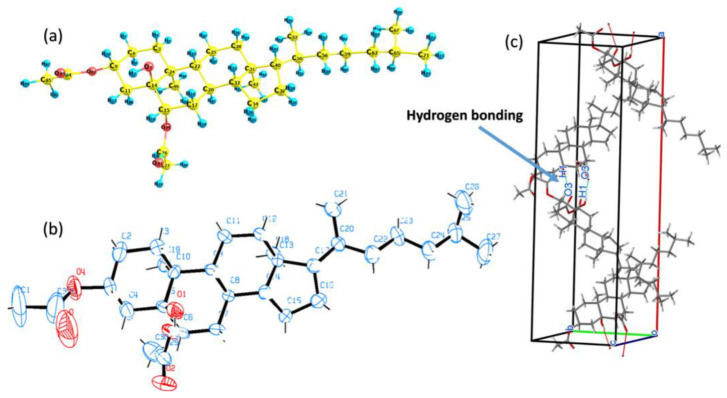
(**a**) Geometry optimization using a B3LYP/6-311++G(d,p) basis set, (**b**) An ORTEP plot of molecule 3, with the atoms numbered at the 50% probability level, and, (**c**) A visualization of the molecular packing of 3, with hydrogen bonds (O-H···O, H···O-H) shown as dashed lines.

X-ray analysis of the synthesized steroid compound confirmed its structure and stereochemistry. 3β,6β-diacetoxy-5-hydroxy-5α-cholestane (3) crystallizes in the space group C2 monoclinic crystal system. The unit cell contained four asymmetric molecules, each with similar geometric parameters. The dimensions of the unit cell are a = 31.634(3), b = 9.9420(11), and c = 9.7325(11). The steroid backbone adopts a chair conformation in the A, B, and C rings [35]. Ring A and ring B contain acetoxy groups at C3 and C6, respectively, while there is a hydroxyl group at C5 at the α position, which connects the junction of the two rings. The anisotropic displacement tensor of the terminal atoms of the 3β-acetoxy group, present at the 3-position of the cholestane skeleton, is strongly anisotropic, indicating that these atoms have a large amplitude of vibration perpendicular to the mean plane of the group, as has been reported in the literature [36]. The five-membered D ring is in an envelope conformation with C13 at the flap. The A/B and C/D ring in the steroid core exhibit trans junctions, which means that the C-C bonds connecting these rings are oriented in a trans configuration with the two carbon atoms on opposite sides of the ring. All of the carbon atoms in the lateral alkyl chain are in a straight line because the chain is fully extended. Steroids are characterized by the presence of trans junctions and a fully extended lateral alkyl chain. These characteristics affect how steroids interact with other molecules, which is crucial for the biological activity of steroids. The average bond lengths are: C(sp3)–C(sp3) = 1.53 Å, C(sp2)=O = 1.23 Å, Csp3–O = 1.45 Å, and Csp3–H = 0.959 Å. These bond lengths are very close to their theoretical values. There are intermolecular hydrogen bond interactions between the C5-O-H···O=C-O-C6 and C3-O-C=O···H-O-C5 groups of the nucleus of steroids (Table 1). These interactions are similar in distance, 2.077 Å, and enrich the stability of the crystal structure by keeping the molecules in a regular array, as shown in packing Figure 1. Some bond distances, especially the acetyl group at C3, were found to be slightly altered. This may be because the crystal quality was not standard quality for diffraction analysis. The endocyclic and torsional bond angles in the steroid skeleton are shown in Table 1. These angles were determined by X-ray crystallography and compared with values obtained by density functional theory (DFT) calculations. The experimental result closely resembles the predicted result, indicating that the synthesized steroid compound’s structure is accurate (Figure 1).

Computational methods are increasingly used to study heterocyclic molecules. DFT can predict structures, energetics, and electronic properties. FMOs play a key role in reactivity, optical properties, and biological activity. Reduced density gradients (RDGs) are a measure of the electron density in a molecule. They are significant because they can be used to identify regions of high electron density, which are often associated with chemical bonds. RDGs can also be used to study the interactions between molecules. To study the electronic structure of the steroid molecule (3), a Gaussian software package was utilized to conduct calculations employing density functional theory (DFT). The initial coordinate source for theoretical calculations was the Crystallographic Information File (CIF). Density functional theory (DFT) was employed to optimize the molecular structure of 3β,6β-diacetoxy-5-hydroxy-5α-cholestane, utilizing the B3LYP functional and the 6-311++G(d,p) basis set. The structures obtained from the DFT calculations are shown in Figure 1a along with the corresponding numbering scheme. The calculated values by DFT calculation were matched with the values achieved by X-ray diffraction analysis. The results demonstrate a correlation between the two sets of data, showing agreement between the electronic structure determined by DFT, the experimental results from X-ray diffraction (Table 1), and the literature [36]. Certain bond lengths and torsional angles, however, showed deviations because the DFT calculations were performed in the gas phase, whereas X-ray diffraction experiments are typically performed on solid-state samples. Furthermore, the molecule’s crystal must be capable of producing accurate diffraction patterns.

The crystal packing of the molecules reveals that they exist as O-H⋯O hydrogen-bonded dimers. The hydrogen bonding occurs between the hydroxyl group at C5 of one molecule and the carbonyl O atom of the acetoxy moiety of the other molecule. The O–H⋯O hydrogen bond distances are 2.81 Å. In addition to the hydrogen bonds, there are also three short intramolecular distances: H30B···O4 2.56 Å, H6···O3 2.65 Å, and H30A···O4 2.56 Å. These short distances suggest that there is a significant interaction between the polar hydrogen and the carbonyl O atoms. The interaction between these atoms helps stabilize the molecule by keeping the atoms in close proximity. This interaction also helps determine the structure of the molecule by affecting the orientation of the atoms (Table 2).

The six-membered rings in a molecule (3) adopt conformations that closely resemble a chair form. This is supported by the puckering parameters determined by Cremer and Pople (1975) [37]. The puckering parameters for ring A are Q = 0.584 Å, θ = 3.6°, and φ = 212°. For ring B, the parameters are Q = 0.549 Å, θ = 104°, and φ = 173.6°. And for ring C, the values are Q = 0.576 Å, θ = 176.7°, and φ = 216°. The D-ring exhibits a twisted conformation around the C13—C14 bond with the puckering parameters Q = 0.452 Å and φ = 113.6°, as shown in Table 3. All rings in the molecule are in a trans-fused configuration. The acetoxy group at C3 is positioned 3β with respect to ring A, while the substituents at ring B are in the 5α and 6β orientations, respectively.

### 2.3. Reduced Density Gradients and Frontier Molecular Orbitals (FMOs)

**Figure 2:**
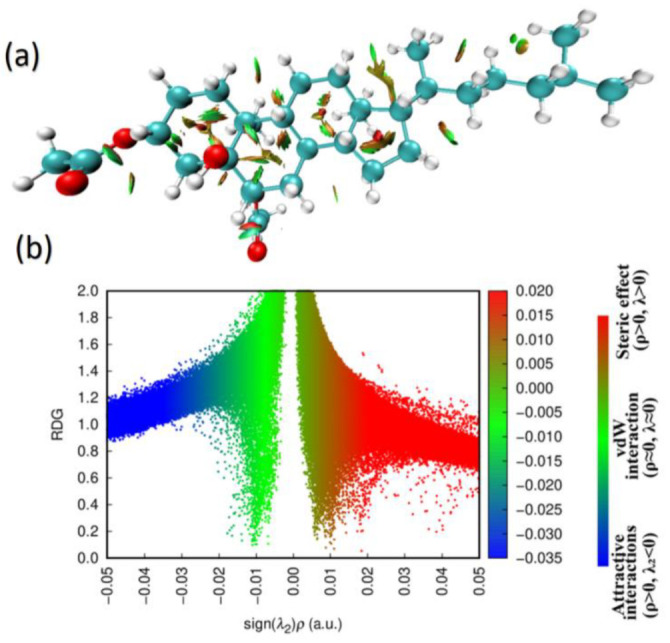
RDG map and NCI plot of the crystal’s gradient isosurface: (**a**) analysis of noncovalent interactions; (**b**) scatter diagram.

Apart from the Hirshfeld surfaces mentioned in Section 2.4, the color-filled reduced density gradient (RDG) of 3β,6β-diacetoxy-5-hydroxy-5α-cholestane was also examined in real space using electron density analysis. This study was performed to further understand the influence of intermolecular and intramolecular interactions within the crystal structure. As shown in Figure 2, different color regions were analyzed to better understand these interactions. The surfaces are color-coded based on the magnitude of sign(λ_2_)p on a scale of blue–green–red. Blue signifies powerful attractive forces, green represents weak attractive interactions, and a strong non-bonded overlap is represented by red in this color scheme. The strength of interactions can be visually assessed by examining the scatter plot and filled isosurface plots. Denser points in the scatter diagram (Figure 2b) indicate a higher electron density and stronger interactions. A dense peak and scattered dots of sign(λ_2_)p within the purview of −0.02∼0 a.u in the provided figure indicate the presence of interactions within the molecule with varying intensities, ranging from weak to strong van der Waals interactions (−0.02 to −0.01 a.u.). The green peak observed only in this region confirms the absence of intramolecular hydrogen bonding within the molecule. Furthermore, the predominance of van der Waals interactions suggests that they are the main force in the system. Positive values of sign(λ_2_)p indicate the presence of steric effects, resulting in red regions observed in the molecule.

**Figure 3:**
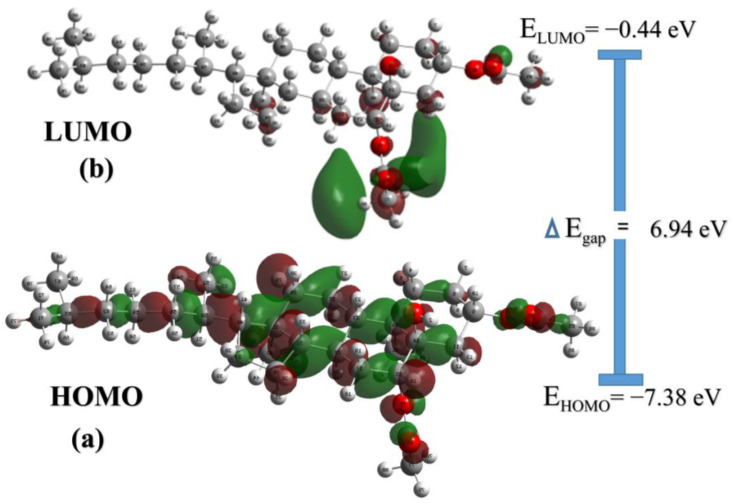
Spatial plots of (**a**) HOMO and (**b**) LUMO showing the energy gap.

The presented data on the frontier molecular orbitals (FMOs) of compound (**3**) are significant, as they reveal key insights into its reactivity, stability, and receptor binding affinity. The molecule’s HOMO and LUMO distribution indicates important binding regions for receptor interactions. Molecular properties are determined by the properties of the frontier molecular orbitals (FMOs), which are the highest occupied molecular orbitals (HOMO) and the lowest unoccupied molecular orbitals (LUMOs). The low-energy LUMO of the acceptor molecule and the higher HOMO value of the donor molecule are both necessary for a molecule to be able to grant electrons to a proper acceptor [38,39]. Molecule reactivity is contingent upon the energy difference between the HOMO and the LUMO, a measurement of the energy disparity between the HOMO and LUMO. A larger HOMO–LUMO gap, which electrons find challenging in transitioning between distant energy levels, indicates lower reactivity. A larger HOMO–LUMO gap means that it is chemically hard because it is difficult to modify it by adding or removing electrons [40]. The spatial arrangement of the HOMO and the LUMO is critical for the recognition of receptor binding sites in ligands (molecules). Figure 3 illustrates that the HOMO and LUMO of 3β,6β-diacetoxy-5-hydroxy-5α-cholestane (3) are mainly distributed on the hydroxyl and acetoxy groups and the steroid ring. This suggests that these specific regions of the ligand are more likely to bind to the receptor, suggesting their importance in receptor interactions. The HOMO and LUMO plots of compound (**3**) are shown in Figure 2, with positive phases highlighted in red and negative phases highlighted in green. The energies HOMO and LUMO are 7.38 and 0.44 eV, respectively, resulting in a 6.94 eV HOMO–LUMO gap. This significant gap reflects the robustness and stability of 3β,6β-diacetoxy-5-hydroxy-5α-cholestane (3), which corresponds to the characteristics of its skeletal structure. The atomic orbitals (AOs) associated with the oxygen atoms of the hydroxy and acetoxy groups located at the 5, 3, and 6-positions of the steroid skeleton (-O-H and -C=O in Figure 1) contribute to the formation of a mixed n- and p-type HOMO and a p-LUMO* when the HOMO and LUMO orbitals of compound (**3**) are surface-analyzed. Consequently, due to the aforementioned interactions, a delocalization effect was noticed in the acetoxy groups when switching from HOMO to LUMO. This means that the electron density spreads out and becomes more distributed over the acetoxy groups in LUMO orbitals compared to HOMO orbitals. A relatively large HOMO–LUMO gap indicates that the compound is chemically hard, which can enhance its stability and receptor binding ability. The delocalized nature of the orbitals may also contribute to these properties. This information can be used to guide the design and optimization of the compound for specific applications, such as receptor binding or drug delivery.

### 2.4. Hirshfeld Surface Analysis

The 3D Hirshfeld surface analysis is crucial for understanding the stability of organic crystals. It quantitatively identifies hydrogen–hydrogen (H-H) interactions as the most significant contributors, comprising 83.8% of the total interactions in the present molecule 3. Specific intra- and intermolecular interactions, which are regarded as the crystal’s support structure, control the stability of organic crystals. Therefore, a crucial component of crystal engineering is the qualitative and quantitative measurement of these interactions. An effective method for assessing the intramolecular and intermolecular interactions that take place during crystal packing is the 3D Hirshfeld surface analysis. Based on a Hirshfeld surface study, two-dimensional fingerprint plots were created using CrystalExplorer 21.5.

**Figure 4:**
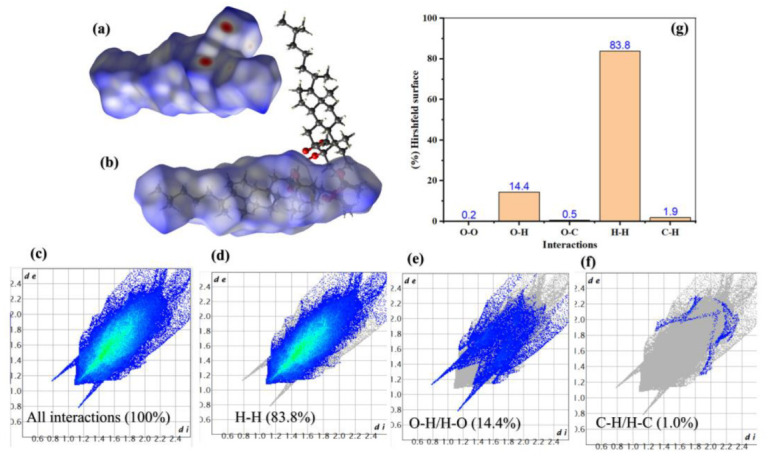
The 2D fingerprint plot shows the distribution of intermolecular interactions in the crystal. The shape index (**a**,**b**) displays the different types of interactions, while the total surface area (**c**) shows the overall interaction network. The proportions of H/H (**d**), O/H/H/O (**e**), C/H/C/H (**f**), and other interactions are also shown in the bar diagram (**g**).

The Hirshfeld surface of steroid (3) was generated with an isovalue of 0.5 using the electron density and mapped onto the dnorm descriptor. The color scheme used ranges from red (shorter distances than the sum of van der Waals radii) to white and blue (longer distances than the sum of van der Waals radii). The 3D mapping was carried out in the range of −0.5293 to 2.1132 dnorm values, where negative values are shown in red, positive values are shown in blue, and zero values are shown in white. These colors represent intermolecular contacts that are short, long, or weak. Intermolecular forces were depicted as crimson circular marks on the Hirshfeld surface. The presence of red circle spots on the pentacyclic ring of steroid (3) in Figure 4a,b indicates intermolecular interactions with a second steroid molecule in a reciprocal manner. Figure 4c–f show a 2D fingerprint plot of 3β,6β-diacetoxy-5-hydroxy-5α-cholestane, which reveals two distinct spikes that display various interactions between neighboring molecules in the crystal lattice. Reciprocal hydrogen–hydrogen (H-H), oxygen–hydrogen (O-H), and carbon–hydrogen (C-H) intermolecular interactions appear in the 2D fingerprint plots as single, double, and unidentified spikes, accounting for 83.8%, 14.4%, and 1.0% of the total plot, respectively. Figure 4d, in particular, shows that H-H interactions are important in maintaining the stability of the crystal structure, as evidenced by their central location among the scattered points. These H-H contacts contribute a significant 83.8% of the total Hirshfeld surfaces, as shown in Figure 4. These findings offer valuable insights in designing new crystals with enhanced properties and predicting crystal packing. The technique also provides qualitative information about the short range and relatively strong nature of H-H interactions, influencing the crystal’s properties. Overall, this analysis aids in comprehending the relationship between the crystal structure and stability, enabling improved crystal engineering.

### 2.5. Human Serum Albumin (HSA)–Steroid Binding Studies

HSA–steroid binding studies investigate the interplays involving human serum albumin and steroids in order to better understand their binding mechanisms. HSA functions as a steroid carrier protein, influencing their bioavailability and pharmacokinetics. Various spectroscopic tools including UV–vis absorption spectroscopy and fluorescence spectroscopy have been employed to investigate the binding interactions between ligands and HSA, allowing for the molecular evaluation of their biological activities. These tools have improved researchers’ understanding of the molecular mechanisms underlying steroid biology and their engagements with human serum albumin (HSA). Moreover, these approaches can be used to identify the participating amino acid residues in steroid binding and judge the binding strength between steroids and HSA.

### 2.6. UV–Vis Absorption Analysis

**Figure 5:**
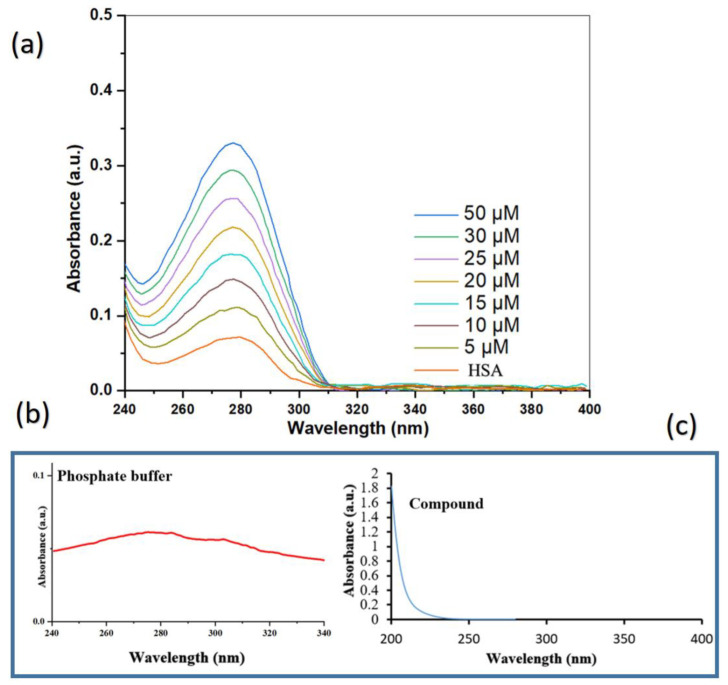
(**a**) HSA–steroid absorption spectra at increasing concentrations of compound (**3**) (5–30 and 50 μM), with the HSA concentration held constant at 5 μM, (**b**) UV–vis spectrum of the buffer, and (**c**) UV spectrum of compound **3**.

UV–visible absorption is a highly responsive method used to observe and study the formation of protein–ligand complexes and gain insight into how complex formation affects protein structural integrity [41]. The procedure is mainly due to the enhanced absorption of UV–vis radiation caused by samples and protein molecules. Tryptophan, tyrosine, and phenylalanine constitute the primary sources of aromatic moieties that are present and absorb light. The maximum wavelength of human serum albumin (HSA) is 280 nm, which is characteristic of the three aromatic residues. Particularly responsive to alterations in the microenvironment, tryptophan absorbs primarily at 280 nm. In general, modifications to the aromatic residues’ microenvironment that follow ligand binding offer insights into the phenomenon of binding. Figure 5 displays the HSA absorption spectra in the presence and absence of 3β,6β-diacetoxy-5-hydroxy-5α-cholestane. The peak in absorbance observed at 278 nm is focused on the microenvironment surrounding the tryptophan and tyrosine residues of HSA. The magnitude of the peaks decreased as the various concentrations of compound **3** were introduced to the constant amount of HSA. Overall, the peak of the magnitude confirms that HSA causes significant structural changes when it interacts with steroids.

To check for interference from the buffer solution, a control was performed with increasing concentrations of the compound ranging from 5 to 50 μM, in the absence of HSA (Figure 5b). The results showed that the buffer solution had a very low absorbance with a flat spectrum at all wavelengths, so it did not interfere with the measurement of the absorbance of other substances. Additionally, no significant change was observed in the UV–vis spectrum of the control as the concentration of steroidal compound **3** was increased. Finally, the wavelength of the compound in the UV–vis spectrum (Figure 5c) was found to be approximately 212 nm, which is outside of the wavelength range of HSA, so there was no interference in the results. 

### 2.7. Fluorescence Studies

**Figure 6:**
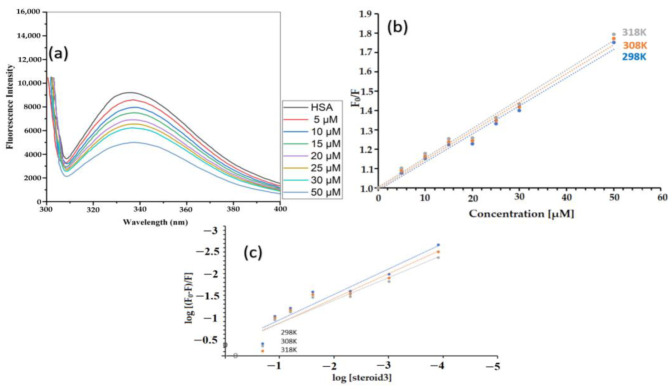
(**a**) Fluorescence emission spectra of HSA–steroid 3 with an increasing steroid concentration (0–30 and 50 μM), while the concentration of HSA was kept fixed to 5 μM in physiological pH 7.4 sodium phosphate buffer at 298 K; (**b**) Stern–Volmer plot of F0/F versus steroid 3 for HSA with steroid 3 interactions at various temperature such as 298 K, 308 K, and 318 K; and (**c**) Modified Stern–Volmer graph plotting log [(F0 − F)/F] vs. log [steroid 3] for HSA with steroid 3 interactions.

Fluorescence spectroscopy has demonstrated its utility as a valuable scientific tool in the investigation of a variety of effects on biomacromolecules, including protein–drug interactions, conformational changes, and the mechanism of quenching in protein–drug interactions [42]. Three amino acids—tryptophan, tyrosine, and phenylalanine—are authorized to regulate the fluorescence of proteins. Tryptophan is the most important fluorophore and fluoresces most strongly in subdomain IIA of the active cavity of HSA. Tyrosine and phenylalanine fluoresce as well, but much more weakly than tryptophan does. Tyrosine fluorescence can be quenched by ionization or by proximity to other amino acids, such as amino groups, carboxyl groups, or tryptophan. In this study, the binding mechanism and formation of complexes involving HSA were explored by titrating it against 3β,6β-diacetoxy-5-hydroxy-5α-cholestane. The concentration of HSA was kept constant at 5 μM, while the concentration of 3β,6β-diacetoxy-5-hydroxy-5α-cholestane was varied from 5 to 50 μM. The presence and absence of the steroid were compared by obtaining HSA spectra, as shown in Figure 6a. The findings indicated that HSA exhibits a fluorescence emission peak at approximately 338 nm. The peak suppression increased as the steroid concentration increased, indicating the formation of a complex that engaged with HSA. The intensity of the fluorescence of HSA dropped by about 41% at the highest steroid dose (50 μM), while the wavelength (λem) and peak morphology remained unaffected. This suggests that steroid 3 binds to HSA in a non-covalent manner. 3β,6β-diacetoxy-5-hydroxy-5α-cholestane (steroid) induced a slow fluorescence quenching in HSA. The quenching of HSA fluorescence by the drug was concentration-dependent, with more prominent quenching observed at 50 μM. The mechanism of fluorescence quenching can take place statically or dynamically. A stable complex between the protein and the quencher results in the quenching of static fluorescence. Various studies consistently demonstrate an inverse relationship between the quenching constant (Kq) and temperature. This relationship is attributed to the fact that the frequency of diffusion decreases with increasing temperature. Dynamic fluorescence quenching occurs due to transient collisions between the quencher and protein. As the frequency of collisions rises, the quenching rate constant (Kq) also escalates in a temperature-dependent manner. Additionally, without actual ligand binding, dynamic fluorescence quenching can take place, which means that HSA’s shape and function are unaffected, providing consistency with the literature. The mechanism of fluorescence quenching was determined using the Stern–Volmer equation establishing a mathematical relationship between the quencher concentration and protein fluorescence quenching. The Stern–Volmer plot demonstrating the interaction of HSA with steroid (3) is shown in Figure 6b. The Stern–Volmer quenching constant (Ksv) can be used to estimate the quenching efficiency of a drug. A higher Ksv value indicates that the drug is better at quenching the fluorescence of a fluorophore (any macromolecule or protein). The Ksv coefficient of the present steroid 3 was determined to be 1.45 × 10^4^ M^−1^. These values indicated that, with a certain amount of the quencher added, the fluorescence intensity decreases by 1%. The bimolecular quenching constant, Kq, can be calculated from the quenching constant, Ksv, and the average time spent in the excited state, τ0, using the following Equation (1):

kq = Ksv/τ0(1)

where Ksv is the Stern–Volmer constant and τ0 is the average lifetime of the protein in the absence of a quencher. For biopolymers, the value of τ0 is usually 10^−9^ s.

The Stern–Volmer equation was used to estimate the rate constant at which HSA and steroid (3) bind, also known as the bimolecular quenching rate constant (Kq). The value of Kq was found to be approximately 1.45 × 10^12^ M^−1^s^−1^. This finding supports the existence of a static quenching process that contributes to the formation of a stable steroid–HSA complex in its resting state. The bimolecular quenching constant, Kq, is necessary to comprehend how HSA and drugs interact. The interaction between HSA and steroid (3) at ambient temperature was studied using a linear regression plot of F0/F against [Q]. The KSV and Kq values were determined to be in the order of 10^4^. This finding suggests that the quenching mechanism between HSA and 3β,6β-diacetoxy-5-hydroxy-5α-cholestane likely began with a static quenching mechanism, as documented in the literature [43]. Additionally, employing a modified Stern–Volmer equation relies on a double logarithmic graph plotting log (F0 F)/F vs. log [Q], the binding constant (Kb) and binding sites (n) on HSA can be ascertained. The slope of the resulting line indicates the number of binding sites (n), while the intercept (ordinate of the origin, shown in Figure 6c) corresponds to the logarithmic value of K_b_ (logK_b_). Fluorescence quenching experiments yielded the HSA–steroid (3) binding and thermodynamic parameters, which are also shown in Table 4. In general, drugs with higher binding constants exhibit stronger binding to receptors than drugs with lower binding constants. The regression equation produced a slope close to one, indicating that, under specific conditions, a single binding site with a high affinity for steroids on HSA was found. The steroid 3 displayed a binding constant of 3.18 × 10^4^ M^−1^, ascertained through experimentation. This high value indicates that 3β,6β-diacetoxy-5-hydroxy-5α-cholestane binds well to HSA. In addition, the calculated Gibbs free energy (ΔG_o_) for the binding of HSA to steroids is −9.86 kcal/mol, and these findings show that the binding process happens spontaneously in nature. The molecular docking resulted in a binding score of −8.6 kcal/mol, suggesting a significant interaction between HSA and steroid 3 because it falls within the range of binding affinities deemed biologically relevant. The experimental and predicted binding fractions agree with each other, further supporting the existence of this interaction.

The effect of temperature on the HSA–Stern–Volmer quenching constants with compound (**3**) was investigated. The studies were performed at 298, 308, and 318 K. As shown in Table 4 and Figure 6b, the K_SV_ values decreased as the temperature increased. This indicates that the quenching mechanism is static, as the interaction between HSA and compound (**3**) is not affected by the increased thermal energy. Similarly, the bimolecular quenching constant (Kq) decreased as the temperature increased from 298 to 318 K, which is consistent with a static quenching mechanism. Ligands bind to proteins through non-covalent interactions, including hydrogen bonding, van der Waals forces, hydrophobic interactions, and electrostatic interactions [43]. The dimensions and signs suggested by Ross and Subramanian are employed for the computation of the thermodynamic parameters (ΔH and ΔS) connected to these interactions [44]. The binding forces are controlled by the thermodynamic variables ΔH and ΔS in protein–ligand binding. While ΔS denotes changes in disorder, ΔH represents the reaction’s heat production or absorption. Positive ΔS denotes increased disorder, and negative ΔH denotes heat release, both of which favor binding. The sum of ΔH and ΔS determines the driving force of the binding, ΔG, with a negative value indicating favorable binding. The following Equations (2)–(4) are used to calculate the thermodynamic parameters:

ΔG° = −RT ln(K)(2)

ΔH° = −TΔS° + ΔG°(3)

ΔS° = ΔG°/T(4)

In this context, ΔG° represents the alteration in free energy, ΔH° indicates a change in enthalpy, ΔS° denotes the change in entropy, R stands for the gas constant (8.314 J/mol/K), T represents the temperature (in Kelvin), and K is the constant.

The slope and intercept of the van’t Hoff plot can be used to calculate the enthalpy change (ΔH°) and entropy change (ΔS°) of a binding reaction, respectively [45]. The values for ΔH°, ΔS°, and the binding free energy (ΔG°) are shown in Table 5. A negative ΔH° indicates that the binding process is exothermic and favors the interaction, indicating that the hydrophobic and hydrogen bond interactions between the drug and human serum albumin are exothermic, releasing heat upon formation. A positive ΔS° indicates that there is more disorder during binding, which is advantageous because it suggests that the interactions between the drug and human serum albumin are not very strong, allowing them to bind in more disordered conformations. The presence of a negative ΔH° and positive ΔS° indicates that hydrogen bonds and hydrophobic interactions are the main binding forces between the drug and human serum albumin. These interactions, despite being weak, are advantageous because they dissipate heat and permit more disordered conformations. Analyzing the data in Table 6 shows that the magnitude of the negative values decreases with an increasing temperature. This observation suggests that as the temperature increases, the spontaneity of the reaction decreases.

### 2.8. Molecular Docking Analysis

**Figure 7:**
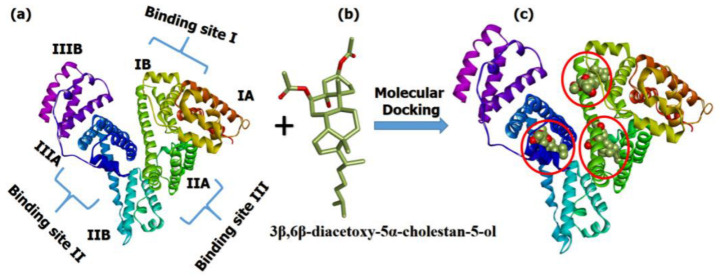
General representation of molecular docking. (**a**) A receptor with three binding sites, labeled I, II, and III. (**b**) Steroid 3. (**c**) Steroid 3 docked in each of the three binding sites.

HSA has three major binding sites referred to as subdomains I, II, and III. These subdomains are allosterically coupled, meaning that the binding of a ligand to one subdomain can affect the binding of ligands to the other subdomains The binding intensity of a compound can vary at different binding sites, such as sites I, II, and III [46]. The best target site in the protein can be predicted based on the binding score obtained from various binding sites in a docking analysis. Molecular docking is a useful computational method for investigating the interactions between macromolecules (like proteins) and small molecules, ligands, or drugs. It plays a crucial role in the drug discovery and development processes by analyzing and forecasting the binding affinities and modes of ligands to target proteins. A popular piece of software called AutoDock Vina uses an algorithm to explore a ligand’s conformational space and forecast the binding pose and affinities it will have for a target protein. By accounting for electrostatic interactions, hydrogen bonds, and van der Waals forces, it evaluates the energy of the interaction between the ligand and the protein. The human serum albumin (HSA) molecule is composed of three structurally related domains: domain I, which contains amino acid residues 1–195, domain II, which contains amino acid residues 196–383, and domain III, which contains amino acid residues 385–585, as shown in Figure 7a. 3β,6β-diacetoxy-5-hydroxy-5α-cholestan, a steroidal molecule (Figure 7b), was individually docked with the active pockets of HSA in domains I, II, and III to examine its binding preferences (Figure 7c). 

**Figure 8:**
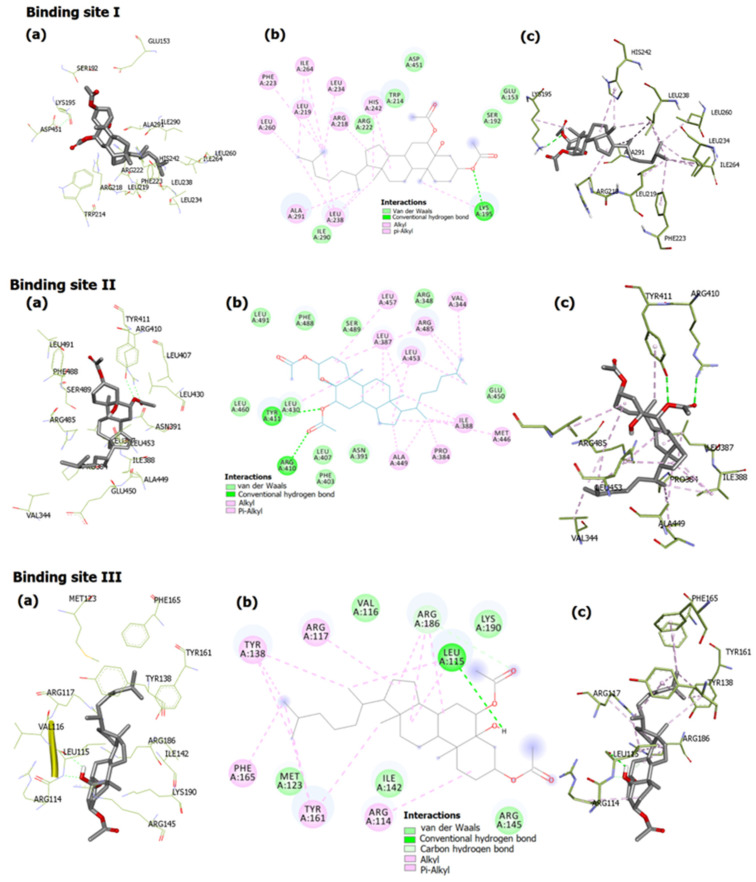
Binding site I, binding site II and binding site III show the best-docked poses with various interactions in (**a**) 3D, (**b**) a 2D-diagram, and (**c**) ligand interactions with active amino acids.

The docked pose of HSA with the steroid (3) in binding sites I, II, and III with the highest binding energy (negative) is shown in Figure 8 (binding sites I, II, and III). 3β,6β-diacetoxy-5-hydroxy-5α-cholestan showed the highest binding energy at three different sites within the HSA. Specifically, the energy of site I was determined to be −8.2 kcal/mol, while it was −8.5 kcal/mol for site II and −8.6 kcal/mol for site III. In the docking model, the presence of actively participating amino acids was observed, contributing to non-bonding interactions such as hydrogen bonding and van der Waals forces at different sites within HSA. To further investigate these interactions, specific docking poses were chosen to analyze non-bonded interactions between steroids and active amino acid residues responsible for forming the active pocket of HSA. At binding site 1, steroid (3) had a binding score of −8.2 kcal/mol. In this model, the oxygen atom of the acetoxy group forms a hydrogen bond with LYS199 of HSA. In addition, several amino acids including ASP451, TRP214, ARG222, GLU153, SER192, and ILE290 were found to surround steroids (2), resulting in van der Waals interactions. This information is depicted in Figure 8. Additionally, salt bridges and hydrophobic interactions between various amino acids and various steroid atoms at particular distances were seen (Table 6). The amino acids LEU115, TYR138, ILE142, TYR161, PHE165, ARG186, HIS146, ARG186, and LYS190 were involved in these interactions. These interactions, along with the hydrophobic interactions and salt bridges including alkyl and pi-alky, collectively contributed to the proper orientation and stabilization of the ligand within the active pocket.

As a result, the close interactions between the ligand and the active amino acid residues yielded a high docking score. The details of these interactions can be found in Table 6.

At binding site II, the best-docked pose of the steroid (3)–HSA obtained by various interactions was found to have the highest binding score of −8.5 kcal/mol. An analysis of Figure 8 (binding site II) shows that the oxygen atom and keto-oxygen atom of the acetoxy group at the 6-position of 3β,6β-diacetoxy-5-hydroxy-5α-cholestane (3) form conventional hydrogen bonds with the amino acids TYR411 and ARG410, respectively. These hydrogen bonds play an important role in close interactions with amino acids present at the active sites of the protein. van der Waals forces were also observed, surrounding the molecule with different types of amino acids, including LEU430, ARG348, LEU460, LEU407, PHE403, ASN391, SER489, PHE488, LEU491, and GLU450. These amino acids, which form non-bonding interactions, assist the molecule and protein in close host–guest interactions. In addition to these interactions, some amino acids, including VAL344, LEU387, PRO384, ILE388, MET446, ALA449, LEU457, ARG453, and LEU453, participate in the formation of alkyl and pi-alkyl interactions of the steroid (3) skeleton, as shown in the figure. Hydrophobic amino acids, such as TRP214, ARG218, LEU219, PHE223, LEU234, LEU238, LEU238, LEU260, ILE264, ILE290, and ALA291, are buried inside the protein such that they are shielded from water. These hydrophobic amino acids make a protein fold stable even during interactions with different atoms of a molecule at specific distances, as shown in Table 6. It was discovered that LYS195 of these amino acids played a role in building a salt bridge with the carboxylate of steroid (3) to increase the stability of the docking model of steroid (3)–HSA.

The best docking model for the steroid (3)–HSA had a binding score of −8.6 kcal/mol, according to the docking simulation. This suggests a strong interaction between site III and the steroid. The OH group of the steroid and the amino acid LEU115 most likely form a hydrogen bond, which accounts for the strong interaction. The steroid’s binding to the site is probably going to be stabilized by this hydrogen bond. In addition to hydrogen bond formation, other non-bonding forces were observed in the best docking model. At binding site III, specific amino acids, namely, VAL116, LYS190, ILE142, MET123, and ARG146, were observed in close proximity to the steroid molecule. These amino acids interacted with each other through van der Waals forces, which are weak attractions between molecules or atoms. ARG186 was also involved in the formation of a carbon–hydrogen bond, indicating that it interacted with a carbon atom in the steroid molecule.

Furthermore, ARG117, TYR138, PHE165, TYR161, and ARG114 displayed alkyl and pi-alkyl forces with the skeleton of the steroid, indicating their contribution to the complex’s stabilization, as depicted in Figure 8. As shown in Table 6, several hydrophobic amino acids, including VAL344, LEU387, ILE388, LEU407, LEU430, ALA499, GLU450, LEU453, ARG485, and PHE488, interacted with different atoms of the steroidal skeleton to improve the docking model’s stability with the protein’s secondary structure. Furthermore, at binding site III, ARG410 and LYS414 formed salt bridges with the steroid’s carboxylate group. This suggests that these amino acids are important for enhancing the stability of the protein–steroid complex. The combined effect of these non-bonding forces results in the overall interaction between the steroid molecule and site III. The strength of this interaction is most likely determined by the number and type of non-bonding forces present. HSA–steroid interactions were examined and compared to previously published interaction profiles for HSA–ligand interactions. The comparison includes various binding sites composed of specific amino acids, such as Asp, Leu, Val, Lys, Phe, Tyr, Ser, Asn, Ala, Glu, Gln, and Gly. Some of these amino acids were also found to be involved in HSA–steroid interactions. These findings are supported by studies conducted by Shen et al. (2017) [47], and Veeralakshmi et al. (2017) [48]. Following a review of the literature, it was observed that a significant number of amino acid residues surrounding drug molecules exhibited hydrophobic properties. This finding suggests that the presence of hydrophobic effects might contribute to the stabilization of complexes formed between drugs and HSA. Likewise, most of the amino acid residues neighboring compound **3** exhibited hydrophobic characteristics. This suggests that the stability of the steroid–HSA complex could be influenced by the hydrophobic effects as well. Molecular docking analyses give a useful framework for observing and investigating on a microscopic level. They enable the quantitative study of the binding affinity score, which can provide insights into ligand–protein interactions [47,48]. The affinity of a compound for binding can exhibit variability based on the structural characteristics of the compound itself, the particular amino acids engaged in the binding process, and the specific binding site involved. As illustrated in this study, compound **3** demonstrates a higher binding affinity towards subdomain IIA compared to its affinities towards subdomains I, II, and III.

### 2.9. Antioxidant Potential Analysis

**Figure 9:**
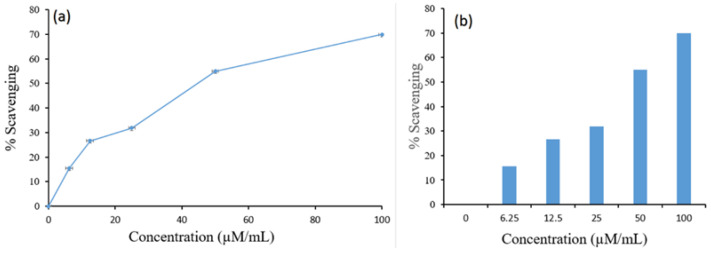
(**a**) DPPH radical scavenging activity (%) versus concentration (μM) and (**b**) bar diagram showing the antioxidant activity.

The ability of steroid 3 to reduce DPPH radicals was used to assess its antioxidant potential. The reduction potential of steroid 3 was measured by a decrease in the absorbance of DPPH radicals at 517 nm. The free radical scavenging activity of steroid 3 may be due to its ability to donate electrons to DPPH free radicals. This steroid has two acetoxy groups at the 3 and 6 positions and an OH group at the 5 position. These functional groups are either electron-rich or readily transfer hydrogen, and they can donate electrons to the unpaired electrons of the DPPH radical. This leads to the formation of hydrazine, a stable molecule with paired electrons. The findings indicated that steroid 3 could lower DPPH radicals, and the IC_50_ value—the amount of steroid 3 needed to lower 50% of the DPPH radicals—was determined to be 58.5 μM. This is shown in Figure 9a,b, which represent the percentage of scavenging versus the concentration. This demonstrates the significant free radical scavenging activity of steroid 3. Although the IC_50_ value of steroid 3 was notably higher than the standard (ascorbic acid IC_50_ = 47.7 mM) [49], careful examination revealed that, in terms of the antioxidant study of samples, the IC_50_ values of the sample and the standard were relatively close. This suggests that steroid 3, albeit to a slightly lesser extent, is also effective at scavenging free radicals. Scavenging free radicals is an important antioxidant activity, so this suggests that compound **3**, a steroid, has the potential to be a promising candidate for the development of novel antioxidant therapies.

## 3. Materials and Method

Melting points are reported uncorrected. Infrared (IR) spectra were obtained by a Perkin Elmer 1600 FTIR spectrophotometer with KBr. Proton and carbon nuclear magnetic resonance (^1^H & ^13^C-NMR) spectra were recorded using a Varian VXR-300s instrument with Me4Si and CDCI_3_ as the internal standard. Chemical shifts are reported in parts per million (ppm), and the abbreviations are used to describe the signals observed (s, singlet; br, broad; m, multiplet centered at; d, doublet; t, triplet). When deuterium (D_2_O) was added, the O-H signals disappeared without significant alterations in other regions of the NMR spectra. Ultraviolet (UV) spectra were captured in chloroform using a Perkin Elmer 1800 FTIR instrument. To visualize spots and track the advancement of reactions, silica gel G-coated TLC plates were employed, along with iodine. Chromatograms were developed using mixtures of light petroleum-benzene and CHC1_3_-acetone. Acetone was used to crystallize the product for further purification, and anhydrous sodium sulfate was employed as a desiccant to dry a solution.

### 3.1. Preparation of 3β,6β-Diacetoxy-5α-cholestan-5-ol

3β,6β-Diacetoxy-5α-cholestan-5-ol can be synthesized using two different methods: conventional and solid-state techniques.

*Conventional procedure* [50,51,52,53]: A mixture of cholestane-3β,5α,6β-triol (500 mg) (or 3β-acetoxy-5α-cholestane-5, 6β-diol as an alternative), anhydrous pyridine (3 mL), and acetic anhydride (2 mL) was subjected to heating in a water bath for a duration of 3 h. Pouring the reaction mixture into 50 g of crushed ice allowed it to cool. The solid was washed thoroughly with water and diluted hydrochloric acid (5%, 2 × 20 mL) to remove any residual pyridine. Further washing with water was performed, then filtered under suction, followed by air-drying, resulting in a yellow solid with a 90% yield. The recrystallization of the solid product using acetone resulted in the formation of crystals, which were identified as 3β,6β-diacetoxy-5α-cholestan-5-ol. The melting point of the crystals was determined to be 167–168 °C, which closely aligns with the reported m.p. of 167 °C, as specified in the literature [54].

**Scheme 1:**
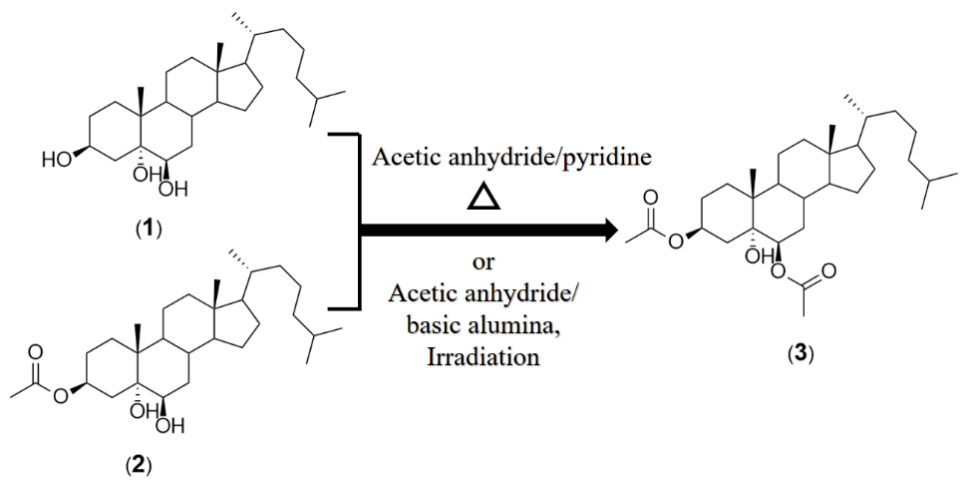
Synthesis of 3β,6β-diethoxy-5α-cholestane-5-ol (**3**). Either cholestane-3β,5α,6β-triol (**1**) or 3β-acetoxy-5α-cholestane-5,6β-diol (**2**) can be used as the reactant, and the synthesis can be carried out using either conventional or solid-state methods.

*Solid-state procedure*: Cholestane-3β,5α,6β-triol (**1**) (250 mg) (or 3β-acetoxy-5α-cholestane-5, 6β-diol (2) as an alternative starting material) (250 mg), acetic anhydride (1.0 mL) or acetyl chloride, and basic alumina (2.0 g) were thoroughly ground using a mortar and pestle. The reaction mixture contained in a 50 mL flask (or vial) was irradiated in a microwave for a duration of 30 s to 2.0 min. The progress of the reaction was monitored using thin-layer chromatography (TLC). The flask was cooled once the reaction was judged to be finished, and the organic materials were then diluted with water and filtered. The precipitate was dissolved in ether, followed by washing with water and drying using anhydrous sodium sulfate. Subsequently, the solvent evaporated, and the resulting residue crystallized from acetone afforded the compound 3β,6β-diacetoxy-5α-cholestan-5-ol (Scheme 1), which exhibited a melting point of 167–168 °C (Scheme 1). Notably, the melting points of the mixtures obtained by the conventional and solid-state synthesis methods were found to be like those of the individual compounds obtained by each method.

Physical, spectral, and analytical data of the synthesized steroid (**3**):

M.p.: 167–168 °C (published m.p. 167 °C); Anal. calc. for C_31_H_52_O_5_: C, 73.77; H, 10.38; Found: C, 73.82; H, 10.33%; IR (KBr, cm^−1^): 3480 (tertiary alcohol, OH, asymmetric), 2931.6–2869.9 (-C–H, symmetric and asymmetric), 1741, 1736, 1266, 1238 for 2 × −OCOCH_3_ (asymmetric and symmetric) 1031 cm^−1^ (C–O–C ether linkage, asymmetric); ^1^HNMR (CDCl_3_, 300 MHz); δ: 5. 37 (1H, dd, C6-αH, equatorial, J = 9.0, 6.0 Hz), 4.9 (1H, m, C3-aH, axial, W1/2 = 17 Hz, A/B ring function trans), 2.24 (1H, s, OH, exchangeable with D_2_O), 2.07, 2.0 (2 × −OCOCH_3_), 1.03 (s, C10-CH_3_), 0.73 (s, C13-CH_3_), 0.97–0.87 ppm for other side chain methyls; ^13^C NMR (CDCl3, 100 MHz); δ: 171.0 −171.2 (2 × −OC=O-), 78.9 (C6), 75.9 (C5), 71.1 (C3), 57.1 (C14), 56.7 (C17), 44.6 (C9), 44.1 (C13), 43.1 (C10), 39.9 (C13), 39.4 (C24), 38.9 (C4), 36.5 (C20), 36.3 (C22), 35.1 (C1), 32.3 (C7), 30.5 (C8), 28.7 (C2), 27.9 (C25), 25.2–25.3 (C15 and C16), 24.1 (C23), 22.6–22.5 (C26/C27), 21.3 (C11), 21.2–20.9 (2 × CH3-COO-), 18.9 (C21), 16.2 (C19), and (C18) 13.3 ppm; UV λmax(methanol) 212 nm; HRMS (ESI): calcd. for C_31_H_52_O_5_ [M + H]^+^: 505.38; Found: 505.38. The IR and NMR spectra of compound **3**, shown in Figures S1–S3, are provided in the supplementary information accompanying this paper.

### 3.2. Single X-ray Crystallography and Computational Details

The diffraction data for crystals were obtained using the APEX2 diffractometer (Bruker–Noius, 2004). Subsequently, cell refinement and data reduction were performed using SAINT (Bruker, 2003) and the SAINT program, respectively. After making necessary corrections to the collected data, the title compound structure was determined using a direct method facilitated by the SHELXT-2015 software [55]. The structure was further refined using least squares with the help of the SHELXL-2015 software [56], which is included in the Olex2 package program [57]. The refinement process involves the anisotropic refinement of all non-hydrogen atoms. Subsequently, hydrogen atoms attached to non-carbon atoms were positioned based on the difference Fourier maps. The remaining hydrogen atoms were treated as rider atoms and refined using the isotropic displacement parameter. Using MERCURY software, diagrams and publication materials were produced [58].

### 3.3. Computational and Molecular Docking Studies

To examine the molecular structure of 3β,6β-diethoxy-5α-cholestan-5-ol, quantum chemical calculations were performed using the Gaussian 09 program [59]. The calculations were carried out in both vacuum and solvent phases using the 6-311G++(d,p) basis set and the B3LYP density functional [60,61]. The optimized structure is identified at the true minimum of the potential energy level, as confirmed by the actual value of the frequency derived at the same theoretical level. At the same theoretical level, the energy gaps of FMOs (HOMO and LUMO) of 3β,6β-diaoetoxy-5α-cholestane-5-ol are obtained. The effectiveness of long-range forces was proved using the Multiwfn program [62]. The calculation and analysis of a number of parameters related to these forces were made simpler by this application. Isosurface visualizations were also produced with the help of the VMD application to give the examined data a visual representation. To determine weak intermolecular interactions in the crystal, CrystalExplorer 21.5 [63] was used to study weak interactions between molecules in 3β,6β-diethoxy-5α-cholestan-5-ol crystals (3) creating Hirshfield surfaces and 2D fingerprints. Molecular docking is a method for predicting the extent to which small molecules bind to proteins. The Lamarckian Genetic Algorithm (LGA) operated in this study to perform an in-silico approach to the binding patterns of 3β, 6β-diacetoxy-5α-cholestan-5-ol with human serum albumin. The three-dimensional (3D) crystal structure of human serum albumin with PDB ID 1H9Z was used as a model of a receptor, and the crystallographic information file (CIF) of 3β, 6β-diacetoxy-5α-cholestan-5-ol was saved in PDF format and used as a ligand. Based on the reported literature regarding multiple active HSA sites [47], three different docking grid boxes were set up, each with a grid spacing of 0.375 and 60 × 60 × 60 points. For binding site I, the box centers were placed at x = 30.025, y = 9.580, z = 10.295; for binding site II, they were placed at x = 12.005, y = 8.737, z = 20.079; and for binding site III, they were placed at x = 33.049, y = 18.888, z = 35.8. The lowest energy structure docked at each active site was selected, and the Discovery Studio was employed to visualize the output and analyze hydrogen bonding and unbound interactions between the receptor and 3β,6β-diacetoxy-5α -cholestan-5-ol. Protein–ligand interaction profiles in multiple binding sites of the Human Serum Albumin (HSA) with steroid 3 were also obtained using the PLIP web tool [64].

### 3.4. HSA-Binding Experiments

#### 3.4.1. HSA Sample Preparation

The concentration of human serum albumin (HSA) for a stock solution prepared by dissolving HSA in a 20 mM phosphate buffer solution at pH 7.4 was determined by measuring the absorbance of an HSA solution at 280 nm using a Perkin–Elmer–Lambda double beam UV–vis spectrophotometer. The extinction coefficient of HSA at 280 nm is 5.30. Sodium dodecyl sulfate-polyacrylamide gel electrophoresis (SDS-PAGE), which revealed a single band, was used to confirm the purity of HSA. To prepare different concentrations of the steroid, a stock solution of 2 mM 3β,6β-diacetoxy-5α-cholestan-5-ol in dimethyl sulfoxide (DMSO) was prepared and diluted in phosphate buffer. Blanks were made using 20 mM PB at pH 7.4 and 298 K. The pH of each solution was determined using an Orion-401-plus pH meter and an Orion glass electrode. The absorption spectra of HSA–steroid complexes were measured at increasing concentrations of the compound (5–50 μM). The concentration of HSA was held constant at 5 μM in this HSA binding study.

#### 3.4.2. UV–Vis Absorption Titration

At a temperature of 298 K, the UV–vis absorption spectrum of HSA was examined. Measurements were performed with and without various concentrations of 3β,6β-diaoetoxy-5α-cholestan-5-ol. The experiment was carried out using quartz cuvettes with a 1 cm path length and a Peltier temperature controller on a Perkin–Elmer–Lambda double-beam UV–vis spectrophotometer. The concentration of human serum albumin was maintained at 5 micromolar in 20 mM sodium phosphate buffer, pH 7.4, and then treated with increasing concentrations of 3β,6β-diacetoxy-5α-cholestan-5-ol for titration. Appropriate values for negative controls were removed from HSA-3β,6β-diacetoxy-5α-cholestan-5-ol readings to eliminate sample absorbance.

#### 3.4.3. Fluorescence Quenching Measurement

The intrinsic fluorescence of HSA was assessed using a Shimadzu fluorescence spectrophotometer RF-5301 equipped with a quartz cuvette and a 1 cm pathlength. The excitation wavelength was set at 295 nm, and emission spectra were recorded between 300 and 400 nm. The excitation and emission slit widths were both set to 5 nm. To explore fluorescence quenching experiments in more depth, The Stern–Volmer Equation (5) was used, as shown in references [65,66,67].

F0/F = Ksv [Q] + 1 = kqτ0 [Q] + 1(5)

The effectiveness of a quencher’s (Q) ability to quench fluorescence is gauged by the Stern–Volmer quenching constant (Ksv). It is determined by the average integrated fluorescence lifetime of the fluorophore (τ0), the molar concentration of the quencher, and the bimolecular rate constant (kq) of the quenching reaction. A tryptophan, τ0, equals roughly 10^−9^ s. The modified Stern-Volmer Equation (6) was used to calculate the binding constants (Kb) and number of binding sites (n) involved in the interaction of HSA samples. The free energy change (ΔG^0^) of the process was determined by analyzing the fluorescence quenching data using Equation (6) and then plugging the results into Equation (7). These calculations facilitate the quantitative assessment of the binding constants (K_b_) and number of binding sites (n) involved in the interaction of HSA samples.

Log (F_0_/F − 1) = log K_b_ + n log [Q] (6)

where F_0_ and F represent the fluorescence intensity in the absence and presence of the quencher (sample), K_b_ represents the binding constant, and n represents the number of binding sites, respectively.

∆G^0^ = −RTlnK_b_ (7)

Using the above equation, the change in free energy (∆G°) can be calculated from the binding constant (Kb) at temperature (T), where R is the universal gas constant (1.987 cal mol^−1^K^−1^).

### 3.5. In Vitro Measurement of Antioxidant Properties Using the DPPH Radical Scavenging Assay

The DPPH assay is a common method for determining a substance’s antioxidant capacity. It assesses a substance’s ability to reduce the stable free radical DPPH [68,69]. The method works by observing the reduction in alcoholic DPPH solutions in the presence of antioxidants that donate hydrogen atoms or electrons. Measure the hydrogen or electron-donating ability of the compound by observing the bleaching of a purple methanolic solution of 2,2-diphenyl-1-picrylhydrazyl (DPPH) using spectrophotometry. The percent inhibition of DPPH is a reliable indicator of the antioxidant capacity of a sample. The larger the inhibition percentage, the stronger the antioxidant activity. The DPPH assay provides a simple and convenient method for evaluating the antioxidant potential of different compounds in vitro. Of note, this assay specifically assessed the compound’s ability to lower DPPH and did not provide a comprehensive measure of its overall antioxidant activity. In this study, the antioxidant activity of steroid 3 was assessed using the stable free radical 2,2-diphenyl-1-picrylhydrazyl (DPPH). Using the conventional DPPH assay, the sample’s capacity to scavenge free radicals was evaluated. For the UV measurements, a freshly made solution of DPPH in methanol (6 × 10^−5^ M) was employed. Steroid 3 solutions were added to the DPPH solution in a 1:1 ratio and vortexed. The concentrations of the Steroid 3 solutions ranged from 6.25 to 100 μM. In other words, 1 mL of the steroid 3 solutions and 1 mL of the DPPH solution were combined. The reaction mixture was thoroughly vortexed and incubated for 30 min at room temperature in the dark. The standard used was ascorbic acid. At 517 nm, the inhibition percentage of the activity to scavenge DPPH radicals was determined using the following equation:

Inhibition (%) = [(A_0_ − A)/A_0_] × 100

A plot of the percentage of DPPH inhibition against the concentration of the sample solutions was used to estimate the IC50 values (the concentration needed to scavenge 50% of the free radical). In this case, A_0_ represents DPPH absorbance in the absence of the steroid, and A represents DPPH absorbance in the presence of the steroid.

### 3.6. Statistical Analysis

The data were analyzed using the mean and standard deviation (SD). The number of individual experiments is indicated by n.

## 4. Conclusions

A cholestane derivative was synthesized using an eco-friendly approach. Its binding to human serum albumin (HSA) was studied using fluorescence spectroscopy, UV–vis spectroscopy, molecular docking, and density functional theory. The results showed spontaneous binding (ΔG = −9.86 kcal/mol) supported by negative binding scores in molecular docking. The HSA–ligand system exhibited static quenching, suggesting conformational changes in the protein upon binding. The molecule’s solid-state packing is governed by dominant H···H, C···H, and O···H interactions, while strong intramolecular interactions were found within the crystal structure through Reduced Density Gradients analysis. This is supported further by the frontier molecular orbital analysis, which revealed a large energy gap of 6.94 eV for the molecule. This indicates that the molecule is relatively stable and unlikely to decompose. The DPPH radical scavenging activity of the studied steroids was comparable to that of standard ascorbic acid. This indicates that the steroid may have antioxidant properties, but more research is needed to determine its mode of action and anticancer activity in vivo. Overall, our findings shed light on the mechanism of cholestane derivative binding to HSA as well as antioxidant analysis. The investigation into the interaction between cholesterol derivatives and human serum albumin (HSA) elucidates a fundamental framework conducive to the advancement of investigations in the domains of pharmaceutical delivery systems, agents for cholesterol reduction, cholesterol metabolism, and the integrity of peptide structures. These findings pave the way for potential future applications in research endeavors. These findings could also have implications for the development of new HSA-targeting therapeutic agents, as well as anticancer research.

## Figures and Tables

**Table 1:** Comparison of some important geometrical parameters of optimized compound **3** with XRD data parameters.

Bond Length (Å)	DFT	XRD	Bond Angle (°)	DFT	XRD	Bond Dihedral (°)	DFT	XRD
C9-O82	1.458	1.434	C11C9O82	109.7313	115.22	C85C84O82C9	178.52	−168.28
O82-C84	1.350	1.430	C6C9O82	107.2475	96.2	C9O82C84O83	−112.4	−46.56
C84-O82	1.208	1.51	C9O82C84	117.6730	102.24	C6C3C24C22	−173.09	−172.70
C84-C85	1.508	1.665	O82C84C85	110.9093	97.0	C6C3C24C46	65.46	66.37
C14-O1	1.449	1.425	C3C24C22	111.0458	109.35	C45C24C22C25	61.91	58.17
O1-H2	0.964	0.821	C3C24C46	108.2078	109.77	C11C14O1H2	48.470	73.57
C9-H10	1.089	0.980	C46C24C22	110.5004	110.14	C11C14C15C17	179.67	178.89
C15-H16	1.092	0.979	C28C31C40	116.4791	118.42	C1C14C15O75	173.30	177.62
C15-O75	1.457	1.456	C28C31C42	110.6404	109.22	C15C14C15O75	−178.32	178.60
O75-C76	1.354	1.314	C42C31C40	110.1862	109.14	C15O75C76O81	1.046	0.81
C76-O81	1.206	1.234	C37C40C50	112.5386	112.93	C25C28C31C40	−165.88	−164.94
C76-C77	1.508	1.506	C40C50C52	113.2645	113.19	C25C28C31C42	67.276	69.37
C77-H80	1.093	0.959	C40C50C56	110.1896	111.27	C42C31C40C58	46.779	48.17
C24-C45	1.548	1.563	C52C50C56	110.3467	110.83	C42C31C40C37	−79.231	−79.66
C31-C42	1.544	1.563	C14O1H2	108.4482	109.44	C37C40C50C52	177.46	176.77
C40-C50	1.549	1.540	C11C14C15	112.0757	113.42	C40C50C56C59	168.60	175.86
C50-C52	1.537	1.523	C14C15O75	109.2216	109.22	C52C50C56C59	−65.573	−55.65
C40-H41	1.099	0.980	C17C15O75	110.9283	109.83	O1C14C24C46	−178.66	−179.90
C50-H51	1.098	0.980	C15O75C76	117.5999	119.15	H33C32C31C42	−177.83	−179.52
C65-C71	1.535	1.523	O75C76C77	110.8554	111.16	C59C62C65C71	−171.31	−169.70
C65-C67	1.535	1.471	O75C76O81	124.0454	122.62	C59C62C65C67	64.6696	69.04
C65-C66	1.099	0.980	C67C75C71	110.4441	109.45	C15C17C28C32	173.10	171.49

**Table 2:** Hydrogen-bond geometry (Å, °).

D—H···A	D—H	H···A	D···A	D—H···A	Symmetry Code
O1—H1···O3	0.78(9)	2.11(9)	2.881(9)	173(12)	x, y, 1−z

**Table 3:** Puckering Parameters of Steroid (3) with Five and Three Six-Membered Rings in its Skeleton.

Cholestane-Skeleton 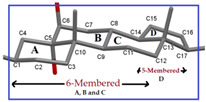	Puckering Parameters		
	Q (Å)	Θ (°)	Φ (°)
Ring A (Six-Membered)	0.584	3.6	212
Ring B (Six-Membered)	0.549	104	173.6
Ring C (Six-Membered)	0.576	176.7	216
Ring D (Five-Membered)	0.452	-	13.6

**Table 4:** In the temperature range from 298 to 318 K, fluorescence quenching experiments were carried out to ascertain the binding and thermodynamic properties of the interaction between has and steroid (3). For this, two equations—the Stern–Volmer equation and the modified Stern–Volmer equation—were used. The steroid concentration ranged from 0 to 30 and 50 μM, while the HSA concentration remained constant at 5 μM.

Temp	Ksv (×10^4^ M^−1^)	Kq (×10^12^ M^−1^s^−1^)	R^2^	K_b_ (×10^4^ M^−1^)	*n*	ΔG° (Kcal/mol)	R^2^
298 K	1.45	1.45	0.9846	3.18	1	−9.86	0.9193
308 K	1.49	1.49	0.9853	2.72	1	−9.53	0.962
318 K	1.5	1.5	0.9855	2.42	1	−9.20	0.985

**Table 5:** Thermodynamic parameters obtained from studying the interaction between a steroid and HSA.

Temperature (K)	ΔG° (kcal/mol)	ΔH° (kJ/mol)	ΔS° (J/mol/K)
298	−9.86	−11.87	20.12
308	−9.53	−11.54	19.72
318	−9.20	−11.21	19.32

**Table 6:** Protein–ligand interaction profiler for hydrophobic, salt bridges, and hydrogen bonding interactions of active amino residues at various binding sites of the Human Serum Albumin (HSA) with steroid **3** were obtained using the PLIP web tool.

Binding Site I									
Hydrophobic Interactions									
Index	Residue	Amino Acid	Distance	Ligand Atom	Protein Atom	Binding Energy (kcal/mol)			
1	115A	LEU	3.93	9098	1736	−8.2			
2	115A	LEU	3.82	9103	1739				
3	115A	LEU	3.64	9119	1737				
4	138A	TYR	3.76	9124	2106				
5	138A	TYR	3.71	9125	2107				
6	142A	ILE	3.30	9103	2183				
7	161A	TYR	3.70	9123	2527				
8	165A	PHE	3.71	9125	2591				
9	186A	ARG	3.70	9109	2896				
Hydrogen Bonds									
Index	Residue	Amino Acid	Distance H-A	Distance D-A	Donor Angle	Protein Donor?	Side Chain	Donor Atom	Acceptor Atom
1	115A	LEU	2.35	2.98	116.15	√	Χ	1080 [Nam]	5609 [O3]
2	115A	LEU	2.19	2.79	118.50	Χ	Χ	5609 [O3]	1083 [O2]
Salt Bridges									
Index	Residue	Amino Acid	Distance Protein	Protein Positive	Ligand Group			Ligand Atoms	
1	146A	HIS	5.36	√	Carboxylate			9126, 9128	
2	186A	ARG	5.00	√	Carboxylate			9130, 9132	
3	190A	LYS	4.21	√	Carboxylate			9130, 9132	
Binding site II									
Hydrophobic Interactions									
Index	Residue	Amino Acid	Distance	Ligand Atom	Protein Atom	Binding Energy (kcal/mol)			
1	214A	TRP	3.28	9108	3327	−8.5			
2	218A	ARG	3.52	9108	3380				
3	219A	LEU	3.45	9125	3405				
4	223A	PHE	3.73	9122	3480				
5	223A	PHE	3.78	9125	3479				
6	234A	LEU	3.53	9125	3651				
7	238A	LEU	3.50	9123	3712				
8	238A	LEU	3.71	9124	3713				
9	260A	LEU	3.44	9124	4020				
10	264A	ILE	3.34	9124	4092				
11	290A	ILE	3.58	9122	4487				
12	291A	ALA	3.38	9119	4504				
Hydrogen Bonds									
Index	Residue	Amino Acid	Distance H-A	Distance D-A	Donor Angle	Protein Donor?	Side Chain	Donor Atom	Acceptor Atom
1	222A	ARG	3.45	4.03	115.90	√	√	3456 [Ng+]	5646 [O3]
Salt Bridges									
Index	Residue	Amino Acid	Distance Protein	Protein Positive	Ligand Group			Ligand Atoms	
1	195A	LYS	4.68	√	Carboxylate			9130, 9132	
2	195A	LYS	4.38	√	Carboxylate			9126, 9128	
Binding Site III									
Hydrophobic Interactions									
Index	Residue	Amino Acid	Distance	Ligand Atom	Protein Atom	Binding Energy (kcal/mol)			
1	344A	VAL	3.39	9124	5311	−8.6			
2	387A	LEU	2.92	9105	5991				
3	387A	LEU	3.14	9103	5993				
4	388A	ILE	3.78	9105	6011				
5	407A	LEU	3.63	9133	6325				
6	430A	LEU	3.54	9133	6711				
7	430A	LEU	3.81	9114	6712				
8	449A	ALA	3.26	9107	7000				
9	450A	GLU	3.73	9123	7011				
10	453A	LEU	3.06	9106	7061				
11	453A	LEU	3.39	9125	7058				
12	485A	ARG	3.53	9124	7547				
13	488A	PHE	3.82	9129	7597				
14	488A	PHE	3.37	9097	7595				
Salt Bridges									
Index	Residue	Amino Acid	Distance Protein	Protein Positive	Ligand Group			Ligand Atoms	
1	410A	ARG	4.77	√	Carboxylate			9130, 9132	
2	414A	LYS	5.26	√	Carboxylate			9126, 9128	

## Data Availability

The findings are supported by experimental data. No further data are available.

## Supplementary Materials

The following supporting information can be downloaded at: https://www.mdpi.com/article/10.3390/molecules28165942/s1, Figure S1: IR spectrum of 3, Figure S2: ^1^H NMR spectrum of 3, Figure S3: ^13^C NMR spectrum of 3, Table S1: Crystal data of titled compound **3**. The supplementary crystallographic data has been deposited at the Cambridge Crystallographic Data Center, CCDC No. 901851 and may be obtained free of charge from http://www.ccdc.cam.ac.uk (accessed on 10 July 2023).

## Sample Availability

Sample is available upon request.
