# Supplementary material for: Exploration of Binding Affinities of a 3β,6β-Diacetoxy-5α-cholestan-5-ol with Human Serum Albumin: Insights from Synthesis, Characterization, Crystal Structure, Antioxidant and Molecular Docking
Source: Molecules. 2023 Aug 8;28(16):5942. doi: 10.3390/molecules28165942 (PMC10459092; doi:10.3390/molecules28165942)
Supplement: Supplementary file 1 [file molecules-28-05942-s001.zip › molecules-2513385-supplementary.pdf]

# Exploration of Binding Affinities of a 3β,6β-Diacetoxy-5α-cholestan-5-ol with Human Serum Albumin: Insights from Synthesis, Characterization, Crystal Structure, Antioxidant and Molecular docking

Table S1. Crystal data of titled compound

| | |
|---|---|
| $C_{31}H_{52}O_5$ | $F(000) = 1112$ |
| $M_r = 504.72$ | $D_x = 1.097$ Mg m$^{-3}$ |
| Monoclinic, *C*2 | Mo *K*α radiation, λ = 0.71073 Å |
| *a* = 31.634 (3) Å | Cell parameters from 8643 reflections |
| *b* = 9.9420 (11) Å | θ = 2.2–24.6° |
| *c* = 9.7325 (11) Å | μ = 0.07 mm$^{-1}$ |
| β = 93.568 (7)° | *T* = 293 K |
| *V* = 3055.0 (6) Å$^3$ | Plate, colourless |
| *Z* = 4 | 0.51 × 0.42 × 0.2 mm |

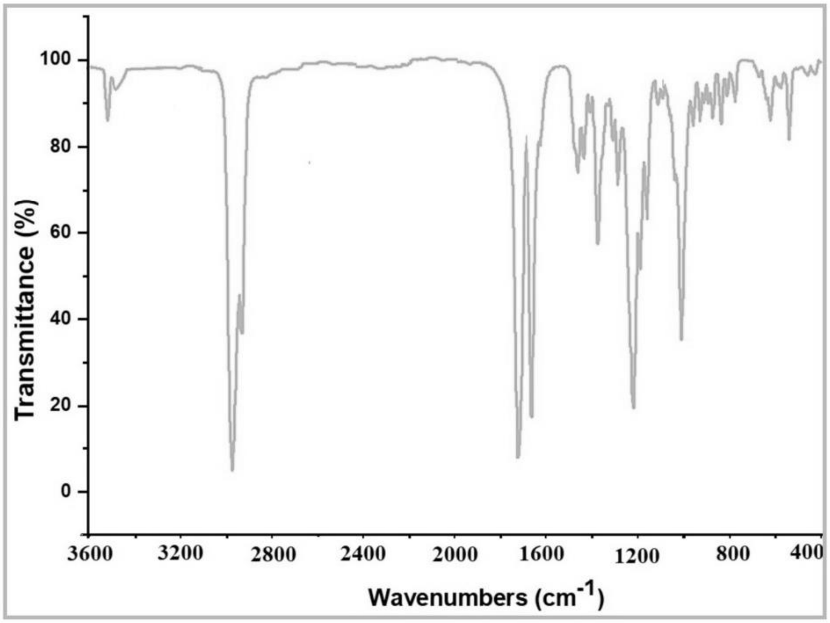


**Figure S1**. IR spectrum of 3

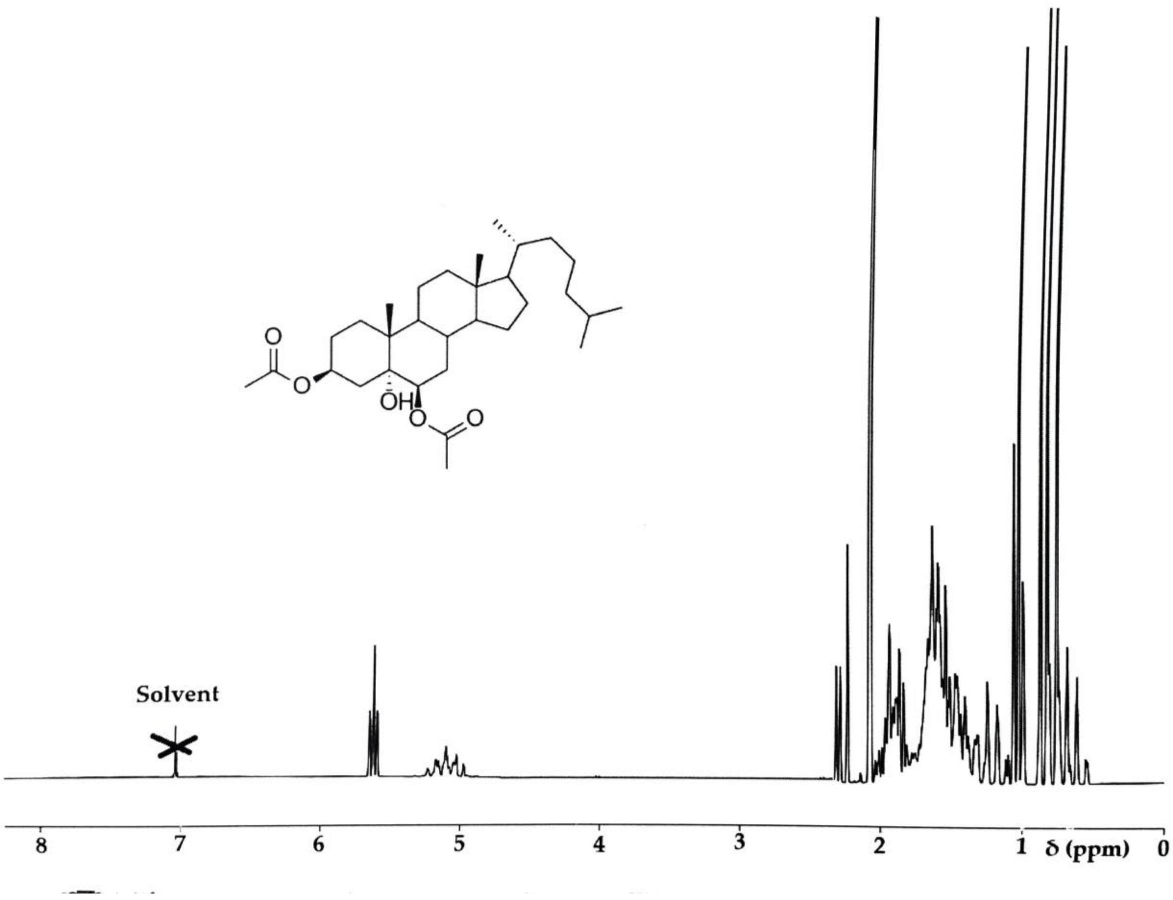


Figure S2. $^{1}$H NMR spectrum of 3

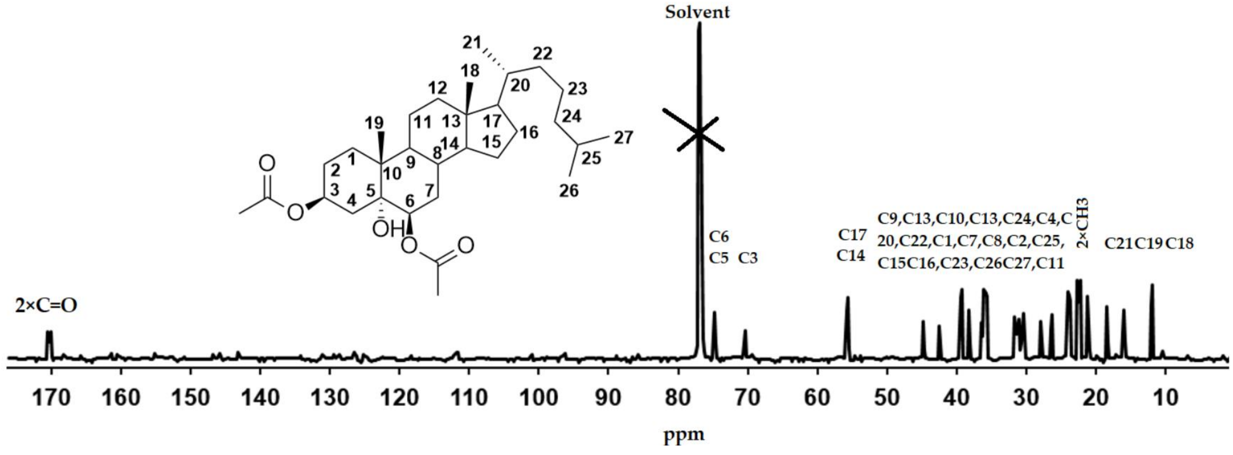


Figure S3. $^{13}$C NMR spectrum of 3
